# A Systematic Review Exploring Variables Related to Bystander Intervention in Sexual Violence Contexts

**DOI:** 10.1177/15248380221079660

**Published:** 2022-03-27

**Authors:** Chelsea Mainwaring, Fiona Gabbert, Adrian J. Scott

**Affiliations:** 1 Department of Psychology, 4898 Goldsmiths, University of London, London, UK

**Keywords:** bystander intervention, social justice ally, sexual violence, systematic review, violence prevention

## Abstract

This article presents a systematic review of the available literature which has investigated the role of key variables in facilitating or inhibiting bystander intervention (including direct intervention, tertiary and secondary prevention) in sexual violence (SV) contexts. Studies exploring the role of individual, situational and contextual variables were grouped to provide a narrative overview of bystanders’ personal characteristics as well as the immediate and wider contexts which may be influencing their bystander behaviour. A systematic search of published literature from four electronic databases identified 2526 articles that were screened, of which 85 studies met the inclusion criteria. Most studies focused upon the role of individual variables, in particular gender of bystander. This body of work finds females are more likely to intervene than males; however, not all studies report these differences and in some cases, this is influenced by the type of intervention behaviour being considered. Regarding situational variables, the most commonly researched variable was the presence of other bystanders, although the role of this variable as inhibiting or facilitating was not clear. Finally, the most commonly researched contextual variable was social norms towards intervention, which has consistently shown greater bystander intervention when there is a belief that peers support such behaviour. Very few studies considered the interaction between these variables. Therefore, it is important for future research to consider this gap in the literature so that we can obtain a more well-rounded understanding of variables that can inhibit and facilitate bystander intervention in SV contexts.

*Sexual violence (SV)* is defined by the World Health Organisation (WHO) as ‘any sexual act, attempt to obtain a sexual act, unwanted sexual comments or advances, or acts to traffic, or otherwise directed, against a person’s sexuality using coercion…’ (Krug et al., 2002, p. 149). SV encompasses a range of different behaviours, including rape, sexual assault, sexual harassment and sexual abuse.

Prevalence rates of SV throughout the world are a great cause for concern. A report published by the WHO found a global prevalence rate of 30% for physical and/or sexual intimate partner violence for women. When looking at women’s experiences of non-partner SV, they found a global prevalence rate of 7% (Garcia-Moreno & Pallitto, 2013). In the US, the National Intimate Partner and Sexual Violence Survey (NISVS) reported that 44% of females experienced some form of contact SV (i.e. touching or penetration) during their lifetime, with 21% reporting completed or attempted rape (Smith et al., 2017). For men, the NISVS reported a prevalence rate of 25%, with 3% having experienced completed or attempted rape (Smith et al., 2017). In the UK, similar findings have been reported with 19% of females having experienced attempted rape and 10% reporting completed rape. Male rates were 5% and 1%, respectively (Macdowall et al., 2013). These figures highlight the pervasiveness of the problem of SV.

Victims of SV experience a range of detrimental physical, emotional, social and psychological impacts (see Basile & Smith, 2011; Walker et al., 2005). The immediate consequences of contact SV include physical injuries as well as long-term gynaecological problems (e.g. Sommers, 2007; Walker et al., 2005). Research shows that victims of SV are more likely to experience poor mental health, with greater post-traumatic stress disorder, anxiety and depressive symptoms compared to those who have not been victims of SV (e.g. Chen et al., 2010; Tarzia et al., 2018). Finally, victims often struggle with social adjustment after the incident, particularly in readjustment in the workplace and in their intimate relationships (e.g. Basile & Smith, 2011; Walker et al., 2005).

The high prevalence and associated impacts of SV have provided the momentum seen in the literature to better understand how best to prevent SV. Prevention efforts are generally categorised as primary, secondary, or tertiary. *Primary prevention* works to deter and inhibit SV before it occurs, by addressing the cultural or structural causes of SV such as personal attitudes, values and beliefs (Larcombe, 2014). Such prevention efforts often take the form of educational programmes or campaigns to address these attitudes, values and beliefs. *Secondary prevention* focuses upon identifying risks and working with groups who are identified as ‘at risk’ of perpetrating SV. Finally, *tertiary prevention* refers to interventions occurring after the event, such as supporting the victim or punishing the perpetrator. One of the main tertiary prevention measures is the involvement of the criminal justice system (Larcombe, 2014).

In addition to the law and educational efforts in preventing SV, engaging with bystanders as a third avenue for prevention has received a significant amount of attention in the literature. *Bystanders* are individuals who witness or are aware of criminal behaviour or social rule violations but are not directly involved in the behaviour itself. They have the opportunity to provide assistance, perpetuate the negative behaviour, or do nothing (Banyard et al., 2005). The bystander literature was originally borne out of wanting to gain an understanding of helping behaviour in emergency and non-emergency situations, and to understand why there seemed to be a reduction in the likelihood of intervention when the number of bystanders increased, known as the ‘bystander effect’ (Latané & Darley, 1968, 1970). The Bystander Intervention Model (Latané & Darley, 1970) was one of the first models put forward to explain the decision making process that bystanders go through when considering whether and how to intervene. The model states that bystanders must notice the situation, perceive the situation as warranting intervention, feel responsible to intervene, have the confidence and skills necessary to intervene and then carry out the intervention behaviour.

Bystander intervention in the context of SV is quite unique in that bystanders can engage in all three types of preventative behaviour (primary, secondary, tertiary), in addition to a fourth type: direct intervention (Powell, 2014). *Direct intervention* refers to behaviour enacted to stop an incident of SV that is occurring in the present. For example, by calling the police or physically confronting a perpetrator during an incident. Bystanders can enact primary prevention behaviour, not in direct response to a SV situation or potential SV situation but in working against the kind of behaviours that encourage and perpetuate attitudes that encourage SV. For example, by challenging a friend who is using sexist language or by taking part in a demonstration or protest aimed at ending SV. Bystander intervention in the context of secondary prevention behaviour includes recognising and then addressing a situation where there is a heighted risk of violence occurring. For example, by informing someone that their drink has been spiked. Tertiary prevention efforts occur after an incident of SV. In the context of bystander intervention, this means supporting a victim or confronting a perpetrator after the incident has occurred. For example, by going with a friend to the police station (Powell, 2014). Such actions are particularly important in the context of SV, as the quality and amount of social support a victim receives plays an important role in their recovery (e.g. Basile & Smith, 2011).

Current efforts in the bystander intervention literature have focused upon gaining an understanding of what variables determine, or are related to, bystander intervention behaviour. This is particularly important for the development of bystander intervention programmes which aim to encourage greater bystander intervention (Banyard, 2008; Banyard et al., 2018). To date, many reviews have considered a wide variety of violent and emergency situations rather than exclusively focusing upon SV. One review which considered the role of contextual factors upon bystander intervention in a range of emergency settings found that social norms, a sense of community, prosocial modelling, policies and accountability cues, and the physical environment, all had an impact on bystander intervention (McMahon, 2015). A qualitative meta-synthesis also showed the importance of peer perceptions in influencing bystander behaviour (Robinson et al., 2020). This review further highlighted the role of individual characteristics of the bystander, such as feelings of responsibility, in addition to other situational characteristics such as the role of alcohol, the presence of peers and behavioural indicators from victims. Similarly, a recent scoping review looked at the barriers and facilitators to bystander intervention in a range of contexts among adolescent bystanders with similar results (Debnam & Mauer, 2021).

The reviews which have been conducted are limited in terms of their applications to SV contexts specifically, with most having focused upon a range of physical and psychological abuse contexts in addition to, or excluding, SV contexts. One systematic review which has focused upon SV contexts found a range of individual (e.g. gender), situational (e.g. relationship between the victim and bystander) and contextual (e.g. peer attitudes) variables to be important to bystander intervention (Labhardt et al., 2017). However, many studies included in the review focused upon both SV and physical violence contexts without evidencing a clear distinction between the two. This limits the certainty in regard to the applicability of these variables in SV contexts. This review also focused solely upon studies which utilised university samples which limits its applications to the general population. Collating the literature from general and student populations will create an even stronger evidence base for the purposes of trying to encourage greater bystander intervention.

As a whole, the limited scope of variables in the available reviews limits our understanding of human behaviour. It has been argued that to sufficiently understand human behaviour, and for prevention efforts to be most successful, it is important to understand the role of individual characteristics and the wider situational and contextual settings in which we function in our everyday lives (Banyard, 2011, 2013). The application of an ecological framework is helpful in rectifying this issue as it aims to move beyond the immediate relationships and environment of an individual to consider the wider context in which an individual exists (Bronfenbrenner, 1977). This review aims to address the limitations of previous reviews and will be the first to provide a systematic and broad understanding of the bystander intervention literature in the context of SV, focusing solely upon direct intervention, tertiary and secondary prevention, and within an ecological framework.

## The Current Study

This article presents a systematic review of the available literature which has investigated the role of key variables in facilitating or inhibiting bystander intervention in the context of SV. We address the question: What individual, situational and contextual variables are related to bystander intervention in SV contexts? There is an accompanying Searchable Systematic Map (SSM) which documents all the individual, situational and contextual variables which have been considered in the literature, and can be viewed on the Open Science Framework (https://osf.io/m2rd4/?view_only=01d17a8b93db4aa39da2b06d370e9a08). The specific aims of this systematic review and the SSM are threefold. First, to provide an overview of the literature regarding what variables have been considered in relation to bystander intervention in SV contexts. Second, to organise this literature within an ecological framework to determine the role of individual, situational and contextual variables in bystander behaviour. Third, to synthesise the findings in regard to the applications and implications for policy, practice and research. The review will provide a clear summary of the most consistent and important findings in the literature and identify current gaps in our understanding which need further attention.

## Method

### Identification

**Figure 1.**
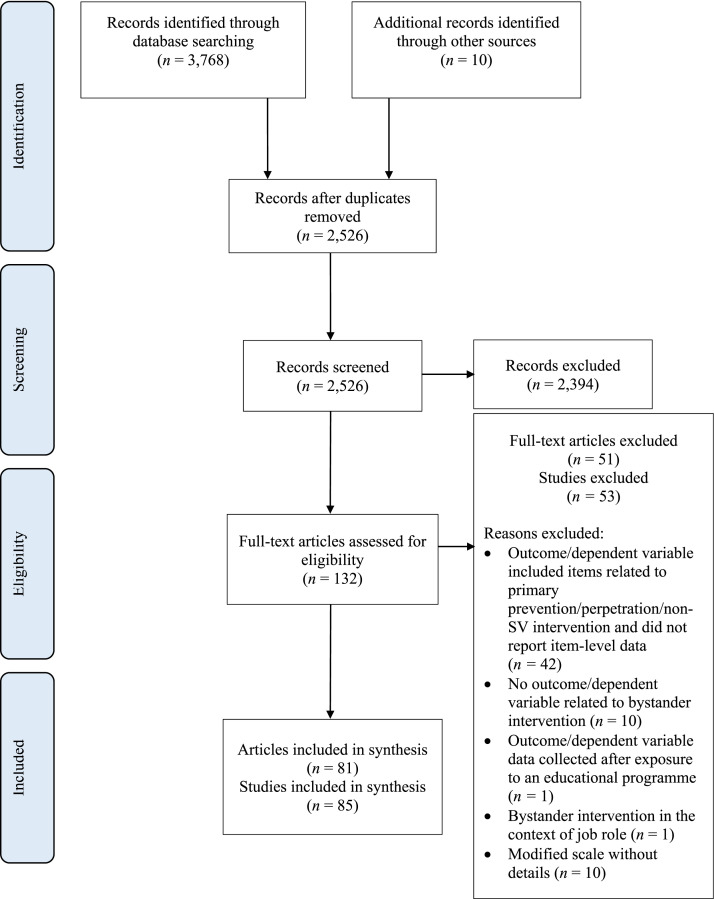
PRISMA flow diagram summarising the literature searching and sifting process.

A search of PsycInfo, Web of Science, Academic Search Complete, and Psychological and Behavioural Sciences Collection databases was conducted in November 2019 to locate published empirical articles. Search terms were refined until all relevant studies from a similar systematic review (Labhardt et al., 2017) appeared in the search results (see Supplemental Appendix A for search terms). In November 2019 and March 2020, ‘hand-search’ steps were taken to identify additional studies. This included searching reference lists of frequently cited articles and ResearchGate profiles of researchers who frequently publish in this field. An additional 10 studies were included at this stage. See Figure 1 for a summary.

### Inclusion and Exclusion Criteria

Studies were included if they examined the relationship between, or effect of, any individual, situational, or contextual variables upon bystander intervention in online and offline SV or unwanted sexual behaviour contexts. ‘Bystander intervention’ in the context of SV or unwanted sexual behaviour could include direct intervention, tertiary prevention, or secondary prevention. For inclusion, ‘bystander intervention’ had to have been reported upon in respect of actual bystander behaviour (either past or present) or willingness/intent to intervene (future), but there were no restrictions in terms of how these behaviours were measured.

Studies were excluded if they: (1) were non-empirical (e.g. literature reviews); (2) published in a non-English language; (3) focused upon evaluating bystander intervention educational materials, programmes, or campaigns; (4) were unpublished, to ensure that the knowledge obtained was peer reviewed; (5) reported upon ‘primary prevention’ behaviours, given they do not relate to actual bystander behaviour for a specific incident of SV and the unmanageable scope of the review should this have been included, with regard to the additional key search terms and number of hits; and (6) looked at bystander intervention in the context of one’s job role, for example, the interventions offered by forensic interviewers upon a victims disclosure of sexual assault. These studies were excluded because individuals are likely to have specific work-based obligations and training to behave in particular ways, which is at odds with how the general population would behave.

### Screening

The screening process was conducted by the primary researcher. First, titles and abstracts of articles were read and assessed against the inclusion and exclusion criteria. Screening was carried out using Zotero using tags to label articles which were excluded at this stage. Articles that met the inclusion criteria progressed to full-text screening. Here, the full text of the articles was read; articles that did not fully satisfy the inclusion criteria or breached the exclusion criteria were excluded from the review. See Figure 1 for the full list of reasons for exclusion at this stage. For both screening stages, if the primary researcher was unsure about the inclusion of any studies, this was discussed further with the Research Team. In total, 81 articles and 85 studies met the inclusion criteria and were included in this review.

### Coding

The extraction of relevant information for all 85 studies was carried out by the primary researcher using MAXQDA. Variables of interest were assigned codes using a bottom-up approach. Study characteristics were predetermined in the SSM and then extracted using a top-down approach. A random sample of 45 studies was checked by an independent researcher to ensure that the study information, methods, measures of variables, outcome variables and analyses were correctly inputted into the SSM. Any conflicts were resolved through discussion between the independent researcher and the primary researcher. To code the individual, situational and contextual variables, the primary researcher created strict definitions and criteria for each variable category. Lower level categories as outlined in the SSM and results that follow were grouped based on ease of interpretation within the review. The following definitions were used to categorise the variables:

- Individual variables reflect individual characteristics and experiences of the bystander. These include: gender, personality, attitudes, or cognitive and emotional processes which occur for that individual. In addition, personal cognitions in regard to bystander intervention in specific situations (e.g. feelings of responsibility) and more generally (e.g. attitudes towards intervention) are classified as individual.
- Situational variables reflect characteristics of the SV incident itself, both in terms of the SV and the people involved. These include: characteristics of the potential perpetrator or victim, relationships among individuals involved, or physical aspects of the space and context at the time of the incident. Characteristics of the situation and those involved which are personal perceptions of the bystanders themselves are also classified as situational.
- Contextual variables reflect characteristics of the wider contextual environment. They are about the bystander’s ‘world’ and the people around them. They reflect the ‘setting’ that the bystander is in. These include: organisational culture or exposure to messages about SV. Characteristics of the context which are personal perceptions of the bystanders themselves are also classified as contextual.

## Results and Discussion

### Overview of Study Characteristics

Of the 85 studies included in this review, the majority were published between 2010 and 2020 (89%) and conducted in the US (84%). Most utilised a university student sample (80%) and the average age was 20.79 years^1^. A quantitative methodology was the most common (79%), with just over half of these studies utilising self-report methods (55%) followed by experimental methods (30%), with the remainder using a combination of methods (15%). Of those using a qualitative methodology, the majority used interviews (38%), followed by focus groups (25%) and written narratives (19%), with the remainder using a combination of methods (19%). Only two studies combined quantitative and qualitative methods (2%). Of those studies using a quantitative methodology, most measured bystander intention (65%), followed by actual bystander behaviour (33%) and only one study measured both (1%). The findings in relation to intent versus actual behaviour will be discussed where relevant, for example, where this distinction helps to explain inconsistencies within the literature.

### Variables Relating to Bystander Intervention

The goal of the current review was to examine the individual, situational and contextual variables that are related to bystander intervention in SV contexts and to consider the application of these variables to policy, practice and research. Findings relating to each variable are summarised and then discussed. Table 1 provides a summary of the critical findings. Table 2 provides an overall summary of the implications of these findings. Given the large number of articles identified for this review, variables that received little attention in the literature and/or produced inconsistent findings are not reported within this review. The variables which have been excluded from this review can be found in Supplemental Appendix B and the SSM and explored further.

**Table 1.** Summary of Critical Findings.

Variable	Critical findings	Example references^a^
Individual variables		
Gender	When differences are found between males and females, generally, female bystanders are more likely to intervene, but this also depends on the type of intervention behaviour. Males are more likely to intervene by confronting a perpetrator, physically interrupting, or choosing an indirect method (e.g. finding someone else to help). Females are more likely to directly intervene with victims and provide post-assault intervention. However, not all studies report differences between males and females	e.g. Franklin et al. (2020); Hoxmeier et al. (2015)
Age	Generally, no effect of age of bystander, although in some cases older bystanders are more likely to intervene. Caution is warranted due to use of restricted samples	e.g. Collazo and Kmec (2019); Hoxmeier, Acock, and Flay (2017)
Feelings of responsibility to intervene	Bystanders are more likely to intervene when they feel greater responsibility to intervene	e.g. Arbeit (2018); Katz, Colbert, et al. (2015)
Confidence to intervene	Bystanders are more likely to intervene when they have greater confidence in their ability to intervene	e.g. Hust et al. (2019); Zelin et al. (2019)
Rape myth attitudes	Bystanders are more likely to intervene when they do not endorse rape myths	e.g. Gable et al. (2017); Zelin et al. (2019)
Previous victimisation	Generally, no effect of previous victimisation upon bystander intervention	e.g. Jacobson and Eaton (2018); Reynolds-Tylus et al. (2019)
Bystander fear of violence	Bystanders are more likely to intervene if they do not fear punitive actions as a result of intervention	e.g. Hoxmeier et al. (2019); Lamb and Attwell (2019)
Situational variables		
Presence of other bystanders	The impact of the presence of other bystanders is unclear. However, bystanders are more likely to intervene when they feel less audience inhibition	e.g. Burn (2009); Katz, Colbert, et al. (2015)
Relationship between the bystander and victim	Generally, bystanders are more likely to intervene if the victim is a friend or known to them. This is due to greater feelings of empathy, responsibility, loyalty and ability to determine whether intervention is warranted	e.g. Franklin et al. (2020); Katz, Pazienza, et al. (2015)
Relationship between the bystander and perpetrator	The impact of relationship between the bystander and perpetrator is unclear. However, interventions which avoid embarrassing the perpetrator are more likely when the perpetrator is a friend or known to the bystander	e.g. Kaya et al. (2019); Wamboldt et al. (2019)
Severity of the sexually violent behaviour	Bystanders are more likely to intervene when the SV is of greater severity	e.g. Bennett et al. (2017); Jacobson and Eaton (2018)
Contextual variables		
Social norms towards intervening against SV	Bystanders are more likely to intervene if there are positive social norms towards intervening	e.g. Reynolds-Tylus et al. (2019); Savage et al. (2017)
Organisational response to SV	Bystanders are more likely to intervene if an organisation exhibits positive or supportive responses towards the handling of SV or the bystander has trust in the handling of incidents of SV within an organisation	e.g. Allnock and Atkinson (2019); Holland et al. (2016)

^a^Additional references can be found in the SSM.

**Table 2.** Implications for Policy, Practice and Research.

Area	Implications
Policy	• Organisations need to have clear policies and practices to handle incidents of SV and ensure that staff and/or students are aware of these and have confidence in the organisation to follow these policies
Practice	• ‘Safe’ bystander intervention options need to be available and potential bystanders need to be aware of these options
	• Bystander intervention programmes should target peer groups given the importance and influence of peer norms
	• Educate about the continuum of SV to ensure that individuals are educated about the severity of all forms
	• Bystander intervention programmes should try to increase feelings of responsibility and confidence in potential bystanders
	• Bystander intervention programmes should try to reduce rape myth attitudes in potential bystanders
Research	• Use an ecological framework when trying to understand what variables are related to or have an effect upon bystander intervention as human behaviour does not exist in a vacuum
	• Need for greater transparency in reporting of self-report measures used, that is, any changes to existing measures need to be reported clearly
	• Need for greater clarity in regard to the bystander intervention behaviour being studied, that is, primary, secondary, tertiary, direct and specific items within any measures used

### Individual Variables

Individual variables are operationalised as variables which are reflective of the characteristics and experiences of the individual bystander. Overall, 72 studies measured, manipulated, or discussed the impact of individual variables upon bystander intervention in SV contexts. The most commonly researched individual variables (based on the number of studies which reported upon these variables) included: *bystander demographics* (including gender and age, *n’s* = 43 and 8, respectively), *bystander cognitions within a SV context* (including feelings of responsibility and confidence to intervene, *n’s* = 11 and 13, respectively), *rape myth attitudes* (*n* = 9), *previous victimisation* (*n* = 9) and *bystander fear of violence* (*n* = 7). These are discussed in turn.

#### Bystander Demographics

The variables of gender and age represent the most researched bystander demographics. In regard to gender, where studies reported a difference between males and females, the majority reported that females showed a greater propensity to intervene compared to males (e.g. Franklin et al., 2020; Savage et al., 2017). However, many studies found no significant differences between males and females in their willingness to intervene (e.g. Banyard et al., 2020; Galdi et al., 2017). Where differences were identified, the majority of the evidence available suggests that female bystanders appear more willing to intervene, although some evidence suggests that this may depend on the type of intervention behaviour. Specifically, males have been shown to be more likely to intervene by confronting a perpetrator, physically interrupting an assault, or by choosing an indirect strategy such as finding someone else to help the victim (e.g. Franklin et al., 2017; Holland et al., 2016). Conversely, females tended to be more likely to directly intervene with the victims during the incident, by either pulling them away from the situation or asking if they are okay (e.g. Holland et al., 2016; Moschella et al., 2018). Females have also been shown to be more likely to intervene post-assault (i.e. supporting the victim after the assault), indicating the use of more tertiary prevention measures (Franklin et al., 2020; Hoxmeier et al., 2015). However, studies have not always found gender differences in regard to different types of intervention behaviour (e.g. Katz & Nguyen, 2016; Palmer et al., 2018) and some studies have found the opposite to be true (e.g. Hoxmeier et al., 2015; Hoxmeier, McMahon, & O’Connor, 2017).

Despite some inconsistency, overall the findings suggest that females take actions that are less risky to their personal safety and focus their attention on the victim rather than the perpetrator. It is also possible that female and male bystanders take different actions due to the gender of the victim and the perpetrator. There is some indication that bystanders feel it is more appropriate to address perpetrators of their own gender (Arbeit, 2018), and given that many sexually violent scenarios involve a male perpetrator and a female victim (Smith et al., 2017), this may explain the distinct actions taken by male and female bystanders in targeting their efforts towards perpetrators and victims respectively. However, additional evidence is needed to determine whether this theory holds.

The differences in female and male actions may explain why some studies fail to find a difference between males and females. Specifically, if outcome measures are constructed using a variety of different types of intervention behaviour, then the differences between males and females may cancel each other out when carrying out statistical analyses with a single outcome variable which is made up of different intervention behaviours. It is also possible that female participants express a willingness to intervene which does not translate into actual behaviour. Nearly all studies which measured actual bystander behaviour did not find any gender differences in regard to likelihood of intervention (e.g. Banyard et al., 2020; Galdi et al., 2017). There were only two exceptions where females were found to have intervened more than males in real-life contexts (Hoxmeier, Acock, & Flay, 2017; Hoxmeier, McMahon, & O’Connor, 2017). Given that inconsistencies remain without a clear understanding as to why suggests that there are mediating or moderating variables at play which need further attention when considering differences between males and females. Altogether, these findings show the importance of considering additional situational variables (e.g. gender of the victim and perpetrator) and the outcome measures which are being used when investigating the role of bystander gender.

With regard to age, many studies found no significant effect of age upon bystander intervention (e.g. Collazo & Kmec, 2019; Hoxmeier, Acock, & Flay, 2017). However, some did find that bystanders who are older are more likely to intervene (e.g. Franklin et al., 2020; Hoxmeier, Acock, & Flay, 2017). The majority of the literature which has considered the role of age has utilised restricted samples (e.g. students) and therefore caution is warranted in concluding whether age has an impact upon bystander intervention.

#### Bystander Cognitions in SV Contexts

The most consistently researched bystander cognitions are feelings of responsibility and confidence to intervene. In terms of responsibility, studies have consistently shown that when bystanders feel greater responsibility to intervene they are more likely to do so (e.g. Arbeit, 2018; Katz, Colbert et al., 2015). Similarly, bystanders who have not intervened when they could have, position themselves as outsiders to the incident and shift the responsibility to others (Lamb & Attwell, 2019).

With regard to feelings of confidence to intervene, studies have consistently shown that those who have greater confidence in their ability to intervene and prevent SV from occurring are more likely to do so (e.g. Hust et al., 2013; Zelin et al., 2019). Relatedly, studies have found that the perceived ease of intervention is significantly associated with the likelihood that bystanders will intervene (Hoxmeier, Flay, et al., 2018; Savage et al., 2017). When included in a regression model with other predictors, only one study found that the perceived success or ease was not significantly associated with intentions to intervene (Collazo & Kmec, 2019). Altogether, the literature leads one to conclude that greater feelings of responsibility and confidence are associated with greater likelihood of intervention.

#### Rape Myth Attitudes

Rape myth attitudes represent stereotypes and false beliefs in regard to the experiences of SV that support victim blaming and minimise experiences of SV (Burt, 1980). The majority of studies have shown that bystanders who endorse rape myths are less likely to intervene (e.g. Gable et al., 2017; Zelin et al., 2019). When included in a regression model with other predictors, only one study found this variable to be unrelated to bystander intervention behaviour (Franklin et al., 2017). The endorsement of rape myths can negatively impact bystander intervention because such beliefs minimise the perceived importance of SV incidents (Arbeit, 2018). Altogether, the literature consistently shows that reduced endorsement of rape myths is associated with greater intervention likelihood.

#### Previous Victimisation

This variable refers to the bystander’s previous victimisation experiences in both SV and other physical violence contexts. The majority of studies have shown that previous victimisation does not impact bystander intervention (e.g. Jacobson & Eaton, 2018; Reynolds-Tylus et al., 2019). Where studies have found a relationship, the direction of this relationship is not consistent. These inconsistencies suggest other variables may be impacting if and how a bystander's victimisation experiences influence their intervention behaviour. One such variable may be whether their past experiences were positive or negative, both in terms of input from bystanders and experiences in engaging with external services. Altogether, the literature suggests that previous victimisation is not a variable with a strong association to bystander intervention. However, given the high prevalence of SV victimisation (see World Health Organization, 2021), it is vitally important that the responses to victims of SV continue to be improved. Not only is this important for the well-being of the victims themselves, but also for their willingness to advise future victims to engage with these services.

#### Bystander Fear of Violence

This variable refers to the bystander’s fear of violence when they are considering whether to intervene. Currently, the role of fear has only been highlighted in qualitative research, but all studies have consistently shown that bystanders are less likely to intervene if they fear getting hurt or injured or if there would be punitive actions against the bystander (e.g. Hoxmeier et al., 2019; Lamb & Attwell, 2019). In such cases, more indirect intervention measures are considered, such as contacting an authority (Salazar et al., 2017). In sum, concerns of safety are important in determining the best action to take in response to SV and can impact if action is taken as well as the type of action.

#### Conclusion and Implications Relating to Individual Variables

The current literature has shown the importance of some individual variables in their impact upon bystander intervention, some of which have important implications for policy, practice and research. First, this review has shown that generally, being female is associated with an increased likelihood of bystander intervention in SV contexts. This aligns with previous reviews for other violent contexts (Debnam & Mauer, 2021; Labhardt et al., 2017). However, the current review has also shown that these differences are not always present, and that in SV contexts, when considering specific actions, male bystanders are more likely to intervene through confronting the perpetrator and seemingly more likely to undertake actions which are more of a risk to their safety in comparison to female bystanders. There are many gendered reasons why there are differences between males and females. For example, research has shown that the endorsement of masculine norms can inform bystander intervention behaviours (e.g. Kaya et al., 2019), which may explain the differences seen between men and women in their willingness to intervene. For the role of bystander gender to have greater impact on policy and practice, future research needs to give greater consideration to the changeable causes of these differences, such as masculine norms. Only then can real progress be made in terms of addressing these inhibiting variables through policy and practice. Equally, given that male bystanders are seemingly more likely to confront perpetrators or engage in actions which put them at greater risk, bystander intervention materials and programmes could incorporate more suitable alternative actions which do not carry such risks. By highlighting the likely safety implications of taking such actions, and offering suitable alternatives, the risk to the bystander can be reduced.

In contrast to the role of bystander gender, the role of feelings of responsibility and confidence, rape myths and bystander fear of violence is much clearer. Notably, both greater feelings of responsibility and confidence to intervene have been shown to increase the likelihood of intervention. Similar findings exist in the broader bystander intervention literature for other contexts among adolescent bystanders (Debnam & Mauer, 2021). These findings have important implications for bystander intervention models and bystander intervention materials. First, both of these variables are important within steps three and four of the five-step Bystander Intervention Model put forward by Latané and Darley (1970). This model describes how greater feelings of responsibility and greater feelings of confidence to intervene will encourage greater likelihood of intervention, and the current evidence provides some support for the applicability of this model to SV contexts. Additionally, given the potential to influence these variables (i.e. we can encourage greater feelings of responsibility and confidence), the current evidence provides support for the inclusion of techniques within bystander intervention programmes which aim to increase feelings of responsibility and the skill sets of bystanders who find themselves in these situations.

Similarly, the literature has shown that a reduced fear of violence and lesser endorsement of rape myths is associated with greater likelihood of bystander intervention. Literature from other violent contexts has shown that intervention is less likely when bystanders fear for their own safety (Debnam & Mauer, 2021; Robinson et al., 2020) and there is greater endorsement of rape myths (Labhardt et al., 2017; Robinson et al., 2020). There are some important implications of these findings. First, it is important that bystanders do not put themselves in harm’s way, and therefore efforts should be made to find and publicise suitable alternatives available for bystanders in cases where there is a risk or fear of violent retaliation. Second, educational programmes can work towards reducing rape myth endorsement which would subsequently increase willingness to intervene. Programmes to date which have incorporated techniques to reduce rape myth acceptance, foster feelings of responsibility and build the necessary skills to intervene have had some success in increasing bystander intervention (e.g. Kettrey et al., 2019).

### Situational Variables

Situational variables are operationalised as variables which are reflective of the SV incident itself, both in terms of the sexually violent behaviour and the people involved. Overall, 49 studies measured, manipulated, or discussed the impact of situational variables upon bystander intervention in contexts of SV. The most commonly researched situational variables (based on the number of studies which reported upon these variables) included: *the presence of other bystanders* (*n* = 17), *the relationship between the bystander, victim* (*n* = 16)*, and perpetrator* (*n* = 14), and *severity of the sexually violent behaviour* (*n* = 13). These are discussed in turn.

#### Presence of Other Bystanders

This variable refers to the presence of other bystanders during the potential SV incident and how this presence impacts bystander intervention. Despite the pervasive idea in the bystander literature that the presence of other bystanders inhibits bystander action through a diffusion of responsibility – the well-known ‘bystander effect’ (Darley & Latané, 1968) – this is not consistently supported by the literature. Some studies have found that the presence of other bystanders inhibits action (e.g. Ball & Wesson, 2017; Katz, 2015), whereas other studies have shown that this can encourage bystander intervention (Harari et al., 1985; Katz, Colbert, et al., 2015).

This lack of consistency in the literature may be due to the role of other variables. For example, audience inhibition, which refers to a fear of looking foolish in front of others (Burn, 2009), may explain why the presence of others can be inhibiting. All studies have shown that where bystanders feel a greater sense of audience inhibition they are less likely to intervene (e.g. Burn, 2009; Katz, Colbert, et al., 2015). Research has also shown that bystanders feel greater comfort when intervening if they see others intervening or have successfully convinced others to intervene too (Oesterle et al., 2018; Reid & Dundes, 2017). However, when an incident is already under the care of relevant authorities or being suitably handled by others, bystanders have not intervened (Hoxmeier et al., 2019; Lamb & Attwell, 2019).

Feelings of safety is another variable that helps explain why the presence of other bystanders can increase the likelihood of intervention, as the fear of physical or violent retaliation may be reduced. Supporting this, studies have shown that being in the presence of peers can encourage intervention as it mitigates any fears about being physically attacked in response to intervening (e.g. Kaya et al., 2019; Oesterle et al., 2018). Having said this, one study found that bystanders may avoid intervention when in the presence of peers due to fear that it might escalate the situation (Hackman et al., 2017). For example, intervening with friends may cause the perpetrator’s friends to retaliate and thereby making the situation worse. Equally, when the perpetrator is surrounded by their peers, this can result in a reluctance to intervene (Reid & Dundes, 2017).

Altogether, the role of the presence of other bystanders remains unclear. However, there is some evidence to suggest that our understanding of the role of this variable requires further scrutiny in terms of potential mediating and moderating variables. The literature to date indicates that audience inhibition and feelings of safety may be two important variables to consider in this endeavour.

#### Relationship Between the Bystander, Victim and Perpetrator

The second most commonly researched situational variable is the relationship between the bystander and the victim. Overall, research has found that, regardless of gender, bystanders who are friends with or know the victim are more willing to intervene (e.g. Franklin et al., 2020; Hackman et al., 2017). Only a few studies have reported no impact of the bystander’s relationship with the victim upon intervention (e.g. Moschella et al., 2018; Zelin et al., 2019). Studies have also shown that bystanders who know the victim or are friends with the victim are more likely to directly intervene and less likely to delegate (find someone else to help) compared to those who do not know the victim (e.g. Kaya et al., 2019; Palmer et al., 2018).

The facilitative role of a personal relationship with the victim is due to increased feelings of empathy, responsibility (Katz, Pazienza et al., 2015), and a sense of loyalty and obligation (Gable et al., 2017). Bystanders who are friends with the victim are also in a better position to assess the situation and determine whether it is problematic and warrants intervention (Oesterle et al., 2018; Pugh et al., 2016). For example, friends are able to provide signals to other friends when they are in trouble (Pugh et al., 2016).

Despite the clear facilitative impact of a relationship with the victim, the impact of the relationship between the bystander and the perpetrator is much less clear. Some studies find no effect of the relationship between the bystander and perpetrator upon bystander intervention (e.g. Franklin et al., 2020; Moschella et al., 2018). However, most research has shown that this relationship does have an impact, but the specific nature of this impact is inconsistent. Some research has shown that bystanders who know the perpetrator are more likely to directly intervene or confront them but are less likely to help the victim or engage with outside resources, such as the police or university campus support (e.g. Bennett et al., 2017; Katz & Nguyen, 2016). Other research has shown that bystanders are less willing to directly confront someone they are friends with as they feel there should be a level of trust for their friends (Butler et al., 2017). Strategies that have been reported to manage this situation is for bystanders to use discrete actions to avoid embarrassing their friends in public, such as pulling them away from the situation or distracting them rather than making a scene (e.g. Kaya et al., 2019; Wamboldt et al., 2019).

Overall, the current literature shows that relationships are important in the context of bystander intervention, and despite a greater lack of clarity for the role of the relationship with the perpetrator, one can conclude that bystanders are more likely to engage in actions which support or protect whomever they are friends with.

#### Severity of Sexually Violent Behaviour

This section groups together all of the ways in which studies have measured or manipulated the severity of the sexually violent behaviour. Despite operationalising and measuring the severity of the behaviour in different ways, the overall message from these studies is clear: bystanders are more likely to intervene when the behaviour is of greater severity (e.g. Bennett et al., 2017; Jacobson & Eaton, 2018). This includes when the immediate danger to the victim is apparent (Oesterle et al., 2018; Pugh et al., 2016), or when the behaviour is perceived to meet the threshold of sexual harassment or an ethical issue (e.g. Bowes-Sperry & Powell, 1999; Collazo & Kmec, 2019). Similarly, bystanders appear to wait for a situation to escalate before intervening, or will avoid intervention if the situation is de-escalating (e.g. Arbeit, 2018; Hoxmeier et al., 2019). Altogether, the evidence suggests there are internal ‘thresholds’ of severity that bystanders use to assess whether they will intervene. Inversely, these thresholds act as a barrier in contexts where behaviour is not considered to be serious enough, or to have escalated sufficiently, for bystander intervention.

#### Conclusion and Implications Relating to Situational Variables

The current literature shows that being friends with or knowing the victim and witnessing more severe behaviour both increase the likelihood that bystanders will intervene in SV contexts. This aligns with previous review articles which found that in a range of bystander contexts, intervention is more likely when members of one’s own peer group is the victim (Debnam & Mauer, 2021; Robinson et al., 2020). Additionally, a relationship with the perpetrator seems to encourage greater intervention, but in these cases, intervention will focus upon that which limits the potential negative repercussions for their friend. When considered together, the role of relationships and behaviour severity both seem to play a role in the perceived ambiguity of the situation. For example, witnessing more severe behaviour is likely to reduce the ambiguity of the situation because it would be clear to the bystander that the situation is one where intervention is necessary, thereby increasing the likelihood of intervention. Equally, having a relationship with the victim has the potential to provide greater insight of whether there is a risk to the victim in that case and increases feelings of bystander responsibility.

Altogether, these findings have important implications for policy and practice. Although relationships cannot be controlled or manipulated in the real world, any potential mediators of this effect can help inform the development of materials to help encourage intervention in contexts where a bystander witnesses a situation to which none of the individuals are known to them. One such promising variable which has been considered here is that of feelings of responsibility. Conversely, the findings regarding the severity of the behaviour is much more easily incorporated into policy and practice. By having policies that educate potential bystanders of the range of behaviours which constitute SV and emphasising the severity of those behaviours that are not always considered to be ‘serious’ in terms of victim and societal impacts, bystanders can learn to address their biases which could then encourage greater intervention behaviour for a wider range of incidents.

Despite the clarity of the previously mentioned variables and the associated implications, there are still gaps in understanding in regard to the presence of other bystanders. Evidence has shown the presence of other bystanders to be both facilitative and inhibitive, which is likely due to mediating or moderating variables which are yet to be properly considered in the literature. Two variables that have been considered here are audience inhibition and feelings of safety. Given that many instances of SV will occur in public settings (e.g. parties and bars), an understanding of how the presence of others impacts the likely help that a victim receives is vital. Equally, as the presence of other bystanders is not something under one’s control, it is important for future research endeavours to consider the role of these variables, which could be controlled or at a minimum, help inform the advice or education provided to potential bystanders if they are to find themselves in such situations.

### Contextual Variables

The third and final group of variables are contextual variables, and these are operationalised as variables which reflect the wider contextual environment. Overall, 42 studies measured, manipulated, or discussed the impact of contextual variables upon bystander intervention. The most commonly researched contextual variables (based on the number of studies which reported upon these variables) included: *social norms towards intervening against SV* (*n* = 14) and *organisational response to SV* (*n* = 7). These are discussed in turn.

#### Social Norms Towards Intervening Against SV

Social norms towards intervening against SV reflect beliefs about whether peers would approve of intervention against SV (injunctive norms) or whether peers would enact intervention behaviour against SV themselves (descriptive norms) (Cialdini et al., 1991). Generally, studies have shown that if bystanders believe that peers would approve of them intervening, or they believe that peers would intervene themselves, they have greater intentions to intervene (e.g. Reynolds-Tylus et al., 2019; Savage et al., 2017). Only two studies found no significant relationship (Hust et al., 2019; Leone & Parrott, 2019).

Social norms which are unsupportive of intervention can impact bystander behaviour due to fears of social disapproval or exclusion for taking such actions, thereby making them less likely to intervene (Allnock & Atkinson, 2019; Reid & Dundes, 2017). Some specific fears that bystanders have reported are those of being labelled a ‘cock-blocker’ or a ‘snitch’ by their peers (e.g. Allnock & Atkinson, 2019; Butler et al., 2017). Altogether, these findings suggest that social norms towards intervention is an important contextual variable to consider, and that fears of an unwelcomed response or disapproval from peers can inhibit bystander intervention.

#### Organisational Response to SV

This variable groups together all the ways in which the response of an organisation or individuals within an organisation have been shown to impact bystander intervention. All the variables, in one way or another, reflect the cultural position of an organisation in regard to their handling of SV, which has been considered in a variety of different ways in the literature. In military, sporting and school contexts, both the anticipated response to poor behaviour or to claims of SV, and the response of those in authority, appear to be important to bystanders. Specifically, research has shown that the anticipation of less negative outcomes to the reporting of, or seeking of mental health services (Allnock & Atkinson, 2019; Holland & Cipriano, 2019), and more positive responses from those in charge, are associated with greater intentions to intervene (e.g. Holland & Cipriano, 2019; Kroshus et al., 2018). Relatedly, evidence has shown that having positive relationships with those in authority can increase the likelihood that a bystander will intervene (Allnock & Atkinson, 2019).

Similar findings are reported when looking at organisational expectations and policies. Specifically, evidence has shown that when bystanders receive greater communication about appropriate behaviour in social settings (Kroshus et al., 2018), and the possible legal or financial consequences that may arise should any form of SV take place (Wamboldt et al., 2019), they are more likely to intervene. Studies have also found that bystanders are more likely to report sexual harassment when companies have a zero-tolerance policy towards sexual harassment, as opposed to a standard policy or no policy at all (Jacobson & Eaton, 2018). Of course, the positive impact of policies and procedures in any setting can only be realised if bystanders have trust that the organisation will appropriately enforce them, and may be reluctant to intervene in cases where they do not have this trust (Allnock & Atkinson, 2019). Bystanders who have a greater sense of trust in an organisation’s sexual assault system are more likely to take some form of action (Holland et al., 2016). Altogether, despite a lack of consistency in the way in which organisational responses are operationalised, one can see that organisational responses towards SV has an impact on bystander intervention.

#### Conclusion and Implications Relating to Contextual Variables

Overall, the clear role of both social norms towards intervention and organisational responses to SV shows the importance of a bystander’s wider peer and community context in bystander intervention. Specifically, more positive social norms towards intervention and more positive organisational responses to SV both increase the likelihood that bystanders will intervene in SV contexts. These findings mirror those found in previous reviews which showed that such peer perceptions and cultures within organisations can impact bystander intervention in a range of emergency settings (e.g. Debnam & Mauer, 2021; Robinson et al., 2020). Overall, these findings have important implications for both policy and practice.

Regarding the role of social norms, these findings show the importance for bystander intervention programmes in targeting peer groups, rather than individuals. Within relevant organisations, efforts should be made to target peer groups and encourage them to participate in bystander intervention programmes as a group rather than on their own. Engaging with groups rather than individuals provides an opportunity for the groups to explore the attitudes of their peers, and relevant educational materials which can target these attitudes can facilitate positive peer-group changes. Literature to date has already shown how interventions which target misperceptions of social norms and allow individuals to discuss social norms can have positive impacts on bystander intervention behaviour (e.g. Orchowski, 2019; Orchowski et al., 2018).

The findings in regard to organisational responses to SV also have important implications for policy. Given that more supportive organisational attitudes towards the prevention of SV and against the perpetration of SV can encourage greater bystander intervention, it is important that organisations (e.g. workplaces, universities and schools) have clear policies and procedures in place for handling such incidents. It is also vital that employees or students are aware of these policies and have confidence and trust in the organisation’s ability to enforce and follow the guidance that is in place. The operationalisation of this variable across the literature has varied a great deal. Equally, the majority of the literature to date has only considered the role of organisational responses upon behavioural intent and not actual bystander behaviour. Therefore, future research needs to have greater consistency in investigating the role of an organisational culture as well as greater consideration of actual bystander behaviour. Until there is greater clarity on the specifics of an organisational culture which are particularly important, as well as confirmation that such variables are impactful for actual bystander behaviour, any specific recommendations for policy and practice are pending.

## General Discussion

The first aim of this systematic review was to provide an overview of the literature which has considered the role of variables in bystander intervention in SV contexts. The second aim was to present the literature in the context of an ecological framework by distinguishing between individual, situational and contextual variables. The final aim of this review was to synthesise findings in regard to applications and implications for policy, practice and research. These applications and implications have been outlined for each of the variables within the results section of this review and are summarised in Table 2.

In this section, we focus on more general implications which can apply to all future research endeavours in this area. First, future research needs to give due attention to the type of bystander intervention behaviour under investigation, in terms of how the behaviours are operationalised, the development of research questions for specific types of bystander intervention behaviour, and in the development of outcome measures. As outlined in the introduction, there are four types of bystander intervention: primary, secondary and tertiary prevention, in addition to direct intervention measures (Powell, 2014). However, the consistency in how these forms of intervention is defined is lacking. Equally, some of the literature did not acknowledge these different forms of intervention at all, and therefore did not give due consideration to important differences in either the development of their research questions or outcome measures. These types of intervention behaviour are vastly different so it stands to reason that some variables may have an impact upon some types of bystander intervention but not others. Furthermore, this lack of consideration is likely one of the causes of inconsistent or null findings across the literature. To address this, the literature needs to agree on key terms to describe the variety of bystander behaviours that can occur in the context of SV, as well as acknowledging these differences in the development of research questions and associated measures. We believe that future research would benefit from utilising the definitions outlined in Powell (2014) and the current review given the breadth and specificity that these definitions allow.

An additional implication in regard to the development of outcome measures is transparency relating to questionnaires and measurement items used in research, including justification of particular items when developing measures. Many articles purported to have modified or revised already existing measures in their study but then failed to detail how and why. In the case of this systematic review, if there were no clear descriptions of the modifications made, one could not be sure whether the modified items were relevant for inclusion, and this led to the exclusion of possibly relevant studies.

In line with the second aim of this review, in using an ecological framework it was important to consider the interactions between variables, both within and outside of their primary groups (i.e. individual, situational, contextual), as well as mediations and moderations (Bronfenbrenner, 1977). Unfortunately, very few studies considered these aspects despite the benefits of doing so. It would help, therefore, to uncover any underlying causes for an effect of, or relationship between, variables which appear to influence bystander intervention behaviour. This would be particularly insightful when looking at variables which cannot be modified if one wants to consider the implications for practice. It would also allow a greater understanding of human behaviour more generally in these contexts. Human behaviour is not determined by any one particular variable but rather is determined by many different variables related to our individual characteristics, the situation we find ourselves in, and the wider context in which we function in our everyday lives (Banyard, 2011).

The consideration of individual characteristics and the wider context and the role these have in human behaviour lends itself to the consideration of diversity. Although the current literature has addressed some issues regarding diversity (e.g. gender and age of the bystander), there remains a lack of consideration of other aspects of diversity such as ethnicity, nationality, or culture. As can be seen within the SSM, the majority of the literature in this field, and therefore the studies included in this review, utilised university student samples in the US which limits the diversity and generalisability of the findings. Equally, the majority of the literature utilised white-majority samples. This is an important limitation of the current literature. Furthermore, perceptions and attitudes towards SV differ greatly across cultures (Kalra & Bhugra, 2013), and the current review has shown the importance of a wider context in impacting bystander behaviour. Equally, in regard to bystander gender, current findings are based on cisgender participants and therefore not representative of transgender or non-binary individuals. Transgender individuals are at a much higher risk of experiencing SV (Coulter et al., 2017) and such experiences are likely to impact bystander behaviour in these contexts. Altogether, individual identities and cultural contexts will likely impact bystander intervention and should be given greater consideration in future research.

### Limitations

The current review has some limitations which relate specifically to the inclusion and exclusion criteria. First, as only published articles were included in this systematic review, it is possible that excluded unpublished articles were relevant, including theses and dissertations. Therefore, this systematic review may be subject to the effects of publication bias. However, this decision was made to ensure that the knowledge obtained was peer reviewed to protect against the inclusion of low-quality studies. Future research may benefit from considering unpublished literature alongside quality assessment criteria to ensure a suitable level of quality is upheld.

Second, it is important to acknowledge the strict exclusion of studies which investigated primary prevention bystander behaviours, either as their main focus or by the inclusion of related questionnaire items, as this limits the scope of the review. Primary prevention behaviour remains an important consideration and avenue for research for the prevention of SV, and the exclusion of such studies from this review should not be taken as an indication of irrelevance. However, given the range and scope of behaviours that could be classified as primary prevention behaviours, the inclusion of these would have significantly burdened the review in terms of the number of additional articles. Primary prevention behaviours also have important differences compared to tertiary, secondary and direct intervention measures, namely, that such actions are not in response to a specific incident of SV. Therefore, including studies which looked at primary prevention behaviour would have reduced the clarity of the review’s narrative, particularly since many articles did not directly specify the type of intervention behaviour being studied.

### Conclusion

This review has provided a summary and synthesis of the most important findings in regard to variables which are related to bystander intervention in SV contexts. These findings were structured through the use of an ecological framework by considering the role of individual, situational and contextual variables to provide a holistic understanding of this behaviour. Many variables have been shown to have an impact on, or relationship with, bystander behaviour, and as such has important implications for policy, practice and future research. This review has shown that the triangulation between individual, situational and contextual variables needs improvement if we are to gain a well-rounded understanding of the behaviour of bystanders, and more specifically, what variables are important in determining the likelihood that bystanders will intervene against SV. To focus on these endeavours will ensure that greater improvements are made in regard to policies and practices in encouraging SV prevention through the engagement of bystanders.

## Supplemental Material

sj-pdf-1-tva-10.1177_15248380221079660 - Supplemental Material for A Systematic Review Exploring Variables Related to Bystander Intervention in Sexual Violence Contexts Click here for additional data file. Supplemental Material, sj-pdf-1-tva-10.1177_15248380221079660 for A Systematic Review Exploring Variables Related to Bystander Intervention in Sexual Violence Contexts by Chelsea Mainwaring, Fiona Gabbert, and Adrian J. Scott in Trauma, Violence, & Abuse

sj-pdf-2-tva-10.1177_15248380221079660 - Supplemental Material for A Systematic Review Exploring Variables Related to Bystander Intervention in Sexual Violence Contexts Click here for additional data file. Supplemental Material, sj-pdf-2-tva-10.1177_15248380221079660 for A Systematic Review Exploring Variables Related to Bystander Intervention in Sexual Violence Contexts by Chelsea Mainwaring, Fiona Gabbert, and Adrian J. Scott in Trauma, Violence, & Abuse

## Note

1. Only 54 studies reported an average sample age.
