# Supplementary material for: A Systematic Review Exploring Variables Related to Bystander Intervention in Sexual Violence Contexts
Source: Trauma Violence Abuse. 2022 Mar 27;24(3):1727–42. doi: 10.1177/15248380221079660 (PMC10240636; doi:10.1177/15248380221079660)
Supplement: sj-pdf-1-tva-10.1177_15248380221079660 - Supplemental Material for A Systematic Review Exploring Variables Related to Bystander Intervention in Sexual Violence Contexts [file sj-pdf-1-tva-10.1177_15248380221079660.pdf]

Appendix A

Type of Search and Search Terms used for Database Searches

Database	Type of search	Search terms
Web of science	Topic	TS=((Bystander$ OR “cyber-bystander$” OR cyberbystander$ OR “helping behavio$r” OR observer$ OR “social justice ally” OR “social justice allies” OR “passer-by” OR prosocial) AND (barrier$ OR inhibit* OR hinder* OR facilitat* OR promote$ OR encourage$ OR ecological* OR context* OR situation* OR individual OR propensit* OR proclivity OR capacity OR intention$ OR attitude$ OR support OR increas* OR decreas* OR positive OR negative OR prevent OR “likel* NEAR/5 interven*” OR “willing* NEAR/5 intervene” OR “willing* NEAR/5 report” OR predictor$ OR correlat* OR “relationship with” OR “relationship between” OR “scenario$”) AND (sexual OR rape OR "indecent NEAR/5 assault$" OR “sex offen?e” OR “sex crime”) NOT (dna OR “bystander cell$”))
PsycInfo, Academic Search Complete, and Psychological and Behavioural Sciences Collection	Default [locates terms within titles, subjects, abstracts, and keywords]	(Bystander* OR “cyber-bystander*” OR cyberbystander* OR “helping behavio#r” OR observer* OR “social justice ally” OR “social justice allies” OR “passer-by” OR prosocial) AND (barrier* OR inhibit* OR hinder* OR facilitat* OR promote* OR encourage* OR ecological* OR context* OR situation* OR individual OR propensit* OR proclivity OR capacity OR intention* OR attitude* OR support OR increas* OR decreas* OR positive OR negative OR prevent OR “likel* N5 interven*” OR “willing* N5 intervene” OR “willing* N5 report” OR predictor* OR correlat* OR “relationship with” OR “relationship between” OR “scenario*”) AND (sexual OR rape OR "indecent N5 assault*" OR “sex offen?e” OR “sex crime”) NOT (dna OR “bystander cell*”)
