# Supplementary material for: A Systematic Review Exploring Variables Related to Bystander Intervention in Sexual Violence Contexts
Source: Trauma Violence Abuse. 2022 Mar 27;24(3):1727–42. doi: 10.1177/15248380221079660 (PMC10240636; doi:10.1177/15248380221079660)
Supplement: sj-pdf-2-tva-10.1177_15248380221079660 - Supplemental Material for A Systematic Review Exploring Variables Related to Bystander Intervention in Sexual Violence Contexts [file sj-pdf-2-tva-10.1177_15248380221079660.pdf]

Appendix B

Variables Excluded from Systematic Review

1. **Individual variables**
    a. Bystander demographics
        i. Race/ethnicity
        ii. Sexual orientation
        iii. Marginalised identity
        iv. Marital status
        v. Class
        vi. Political orientation
        vii. Endorsement of a group identity (a typical student on campus)
        viii. Family income
    b. Bystander cognitions in a sexual violence context
        i. Failure to notice potential bystander situation
        ii. Distractedness in potential bystander situation
        iii. Identifying behaviour as problematic or risky
        iv. Perceived appropriateness of intervention
        v. Willingness to intervene in potential bystander situations
    c. General attitudes and beliefs
        i. Exploitative entitlement
        ii. Perception of women
        iii. Attitudes towards racial injustice
        iv. Sexism attitudes
        v. Endorsement of masculine ideology

vi. Masculine gender role stress

vii. Attitudes towards racism

viii. In-group bias

ix. Diversity beliefs

x. Heterosexual beliefs

xi. Hostility towards women

xii. General attitudes towards victims

xiii. Alcohol expectancies

xiv. Endorsement of gendered stereotypes/assumptions

xv. Ethical ideology (relativism)

xvi. Ethical ideology (idealism)

xvii. Morality

xviii. Friendship prioritisation

xix. Confidentiality prioritisation

xx. Individual judgement prioritisation

xxi. Desire to prevent further incidents of sexual violence

d. Personal experience or knowledge of sexual violence

i. Know someone who has been a victim

ii. Personal previous perpetration

iii. Sexual assault awareness

e. Attitudes towards intervention

i. Attitudes toward intervention if they were to intervene

ii. Attitudes about helpfulness of intervention behaviour

iii. Attitudes about personal benefits to intervention

iv. Attitudes about societal benefits to intervention

    v. Attitudes about importance and consequences of intervention behaviour
    vi. Attitudes towards decisional balance
    vii. Outcome expectations towards intervention (positive/negative)

f. Personality
    i. Extroversion
    ii. Self-esteem
    iii. Expressivity
    iv. Instrumentality
    v. Social desirability
    vi. Sexual assault perpetration propensity
    vii. Civil courage
    viii. Personal reputational concern

g. Previous bystander behaviour
    i. Previous sexual violence intervention behaviour
    ii. Previous opportunities for sexual violence intervention

h. Mental health/substance use
    i. Lifetime depression
    ii. Lifetime PTSD
    iii. Use of mental health services
    iv. Alcohol use
    v. Substance abuse
    vi. Binge drinking
    vii. Marijuana use
    viii. Illicit drug use

2. **Situational variables**

   a. Presence of other bystanders

      i. Size of group of bystanders

      ii. Action/inaction of other bystanders

   b. Relationships between the bystander, victim, and perpetrator

      i. Harasser holding power/authority over bystander

      ii. Relationship between victim and perpetrator

      iii. Sex pairing between bystander and victim/perpetrator

      iv. Bystander relationship with other bystanders

      v. Interpersonal relationships

   c. Characteristics of victim

      i. Gender

      ii. Sexuality

      iii. Race

      iv. Promiscuity

      v. Sexual behaviour if they were sober

      vi. Workplace performance

      vii. Clothing

   d. Characteristics of perpetrator

      i. Gender

      ii. Reputation

      iii. Workplace performance

   e. Victim blaming and empathy

      i. Victim blame

      ii. Victim pleasure

    iii. Empathetic concern for victim
    iv. Victim sympathy-worthiness

f. Perpetrator and victim behaviour
    i. Drugging/drink-feeding victim
    ii. Perpetrator took victim away (out of sight)
    iii. Excessive touching from perpetrator
    iv. Distinguishing a perpetrator from a guardian
    v. Perpetrator violating personal space
    vi. Perpetrator turned away by other women
    vii. Perpetrator body language
    viii. Perpetrator persistence in pursuing the victim
    ix. Pursuit of an unconscious victim
    x. Victim reaction

g. Intoxication of individuals
    i. Intoxication of victim
    ii. Intoxication of bystander
    iii. Discrepancy of intoxication between victim and perpetrator

h. Other characteristics of incident
    i. Uncertainty surrounding consent
    ii. Inability to directly intervene due to physical space
    iii. Length of time that had passed since incident
    iv. Visual information available to bystanders
    v. Perceived realism of high risk context
    vi. Familiarity with high risk context
    vii. Anonymity of bystander

    viii. Ambiguity of situation

  i. Type of sexual violence behaviour/incident

    i. Type of sexual harassment
    ii. Type of risk situation

3. Contextual variables

  a. Social norms

    i. Social consensus of what is considered to be sexual harassment
    ii. Injunctive norms towards sexual violence perpetration
    iii. Descriptive norms towards sexual violence perpetration
    iv. Descriptive norms towards misogynistic behaviour
    v. Normalisation of sexual harm
    vi. Gendered understandings of sexual assault
    vii. Injunctive norms towards bother over potential sexual abuse victims
    viii. Misperception of injunctive norms towards bother over potential sexual abuse victims
    ix. Hook-up culture
    x. Sexualisation of work environment
    xi. Cultural norms linking men's social status to sexual activity
    xii. Male peer approval

  b. Media exposure

    i. Objectification of women in the media
    ii. Sports media exposure
    iii. Consumption of men's magazines
    iv. Frequency of watching crime dramas
    v. Consumption of women's magazines

c. Education about sexual violence
    i. Exposure to messages about sexual violence on campus
    ii. Sexual assault training/education exposure
    iii. Accessibility barriers to reporting
    iv. Accessibility barriers to accessing support
    v. Awareness of sexual assault resources available
d. Diversity and inclusion
    i. Intergroup contact frequency
    ii. Intergroup contact quality
    iii. Anxiety about intergroup contact
    iv. Organisational culture towards diversity and inclusion
e. Career/education
    i. Employment status
    ii. Managerial status
    iii. Military branch
    iv. Military rank
    v. Educational experience
    vi. Living in campus housing
f. Social/club membership
    i. Fraternity/sorority membership
    ii. Athletic membership
    iii. Contact sport membership
    iv. Sports division of competition
    v. Sports team bond
g. Military culture/values

i. Army norms/values related to communication and teamwork

ii. Military morale

iii. Belief that sexual assault is a problem in the military

h. Community value

i. Community cohesion

ii. Collective efficacy to make the community safer
